# Gene-Family Extension Measures and Correlations

**DOI:** 10.3390/life6030030

**Published:** 2016-08-03

**Authors:** Gon Carmi, Alexander Bolshoy

**Affiliations:** Department of Evolutionary and Environmental Biology, University of Haifa, Haifa 3498838, Israel; goncarmi@gmail.com

**Keywords:** number of paralogs, comparative genomics, combinatorial optimization, Mycoplasmas, Halophiles, Orientia, *Mycobacterium leprae*, genome size

## Abstract

The existence of multiple copies of genes is a well-known phenomenon. A gene family is a set of sufficiently similar genes, formed by gene duplication. In earlier works conducted on a limited number of completely sequenced and annotated genomes it was found that size of gene family and size of genome are positively correlated. Additionally, it was found that several atypical microbes deviated from the observed general trend. In this study, we reexamined these associations on a larger dataset consisting of 1484 prokaryotic genomes and using several ranking approaches. We applied ranking methods in such a way that genomes with lower numbers of gene copies would have lower rank. Until now only simple ranking methods were used; we applied the Kemeny optimal aggregation approach as well. Regression and correlation analysis were utilized in order to accurately quantify and characterize the relationships between measures of paralog indices and genome size. In addition, boxplot analysis was employed as a method for outlier detection. We found that, in general, all paralog indexes positively correlate with an increase of genome size. As expected, different groups of atypical prokaryotic genomes were found for different types of paralog quantities. Mycoplasmataceae and Halobacteria appeared to be among the most interesting candidates for further research of evolution through gene duplication.

## 1. Introduction

The existence of significant gene redundancy—or, in other words, the existence of multiple copies of protein-coding genes—has been known for a long time. The availability of numerous prokaryotic complete genome sequences confirmed this and provided data to examine various possible factors affecting attributes of gene-families [1,2,3,4]. There are several very fundamental questions related to the origin and variability of gene copy number. In this study, we do not pretend to contribute anything substantial to discussions around above-mentioned fundamental questions. Our work is specifically concerned with association between number of gene copies and genome size. As a rule, we use the term “gene copy” in the study; however, sometimes, we use the term “paralogs” as shorthand for “members of a gene family” or, simply, gene copies. In literature, one can find different usages of the term “paralog” [3,5]. Walter Fitch introduced this essential term [6] bearing in mind the following: paralogs are homologous genes that have diverged from each other because of genetic duplication. We hope that the occasional use of the term will not confuse the reader.

Strictly, a gene family is a set of several similar genes, formed by duplication of an original gene. In this study, for all practical purposes, a *gene family* is a subset of protein-coding genes belonging both to the same clusters of orthologous groups (COG) [7,8,9,10] and to the same genome. Our admittedly oversimplified approach has obvious limitations, yet, statistically it works as well as other more rigorous methods of paralog characterization.

Gene-families (see our operational definition above) are of variable size and of varying degree of similarity among their members. We believe that many aspects of gene-family’s attributes and origins require further study. In this study, we concentrate on the gene-family’s attributes, rather than their origins. Specifically, we do not try to distinguish effects of different types of gene duplication and horizontal gene transfer (HGT), since the relative contribution of gene duplication and HGT to genome expansion and variability is unknown [11,12,13,14].

One of the major associations related to gene-family size is that the latter correlates well with a genome size [11,15,16]. Pushker et al. [4] determined these correlations for 127 eubacterial genomes, updating the earlier work of King Jordan et al., which was done on a more limited dataset [3].

Gene duplication and HGT are the processes that can change the size of numerous gene-families, which is manifested as a discriminating attribute even between different strains of microbes. Expansion of gene-families represents an increased cost for a prokaryote. So, what is the evolutionary driving force behind retention of a gene duplicate? A plausible answer to the question has been proposed: the adaptation to altered environments. The duplicated genes may serve as genetic reservoir for coping with fluctuating environmental conditions such as altered salinity or thermal stress [17]. For the gene copy to avoid deletion, it must represent a positive response to environmental stress, e.g., by just increasing gene dosage as a response to higher demand [11,18]. When the selective pressure is removed, the paralogs may be lost again [17].

What is the role of phylogeny in the process? Pushker et al. [4] wrote: “The relative contribution of these genes [paralogous genes] in each genome seems to be independent of phylogenetic affiliation” referring in support of the statement to [3]. Actually, King Jordan et al., wrote: “… the graph topology recovered from the data on lineage-specific gene expansions reflects a combined effect of phylogenetic relationships, common patterns of gene loss, and horizontal transfer” [3]. A big evolutionary question is whether gene duplication is a random or regulated process. There is an additional question: if a new paralog must evolve to provide a new selectable function, by which gradual evolutionary process would the copy be preserved?

Our study has several goals: (i) to confirm that number of gene copies positively correlates with genome size and to measure the correlation using the biggest available dataset of prokaryotic genomes; (ii) to present quantitative descriptions of gene-family size genome size association; (iii) to use boxplot analysis for outlier detection; and (iv) to find taxa that have atypical associations between gene-family size and genome size, which make them good candidates for further genomic studies.

## 2. Methods

### 2.1. COGs Database and Input for Ranking

Here we used a very simple approach to consideration of paralogs: a gene family is a set of protein-coding genes from the same genome and from the same cluster of orthologous groups. In other words, we used the database of clusters of COGs [7,8,9,10] in order to prepare an input matrix of numbers of gene copies, from which estimates of gene-family extension level (GFE level) are calculated. Historically, information about completely sequenced and annotated prokaryotic genomes was stored at ftp://ftp.ncbi.nih.gov/genomes/, including tables of protein features, called PTT files. On 2 December 2015, the collection was moved to ftp://ftp.ncbi.nih.gov/genomes/archive/old_refseq/Bacteria/. More than 2000 prokaryotic genomes belong to this frozen collection; however, only part of the collection was COG-annotated. So, only those complete and COG-annotated genomes that were included in NCBI dataset were considered. There are 1370 Bacterial and 114 Archaeal complete and COG-annotated genomes in our dataset. Proteins of these genomes are distributed among about 5600 COGs.

We created a combined matrix from this dataset of 1484 prokaryotic genomes. Rows and columns correspond to genomes and COGs respectively. We indexed genomes, thus, the *i*th genome corresponds to the *i*th row of the matrix. Every COG has its NCBI index. Datum in entry (*i*,*j*) is the number of genes from the *i*th genome belonging to the *j*th COG.

The goal was to rank genomes in such a way that genomes with lower number of paralogs would have lower rank. Meaning of the expression “lower number of paralogs” is rather undefined and can be interpreted in several ways. Even defining an optimal ranking is a nontrivial task. In our review [19] we described several approaches to find a nearly optimal ranking using methods from the field of combinatorial optimization. Until now, rank aggregation methods have not been applied to the problem.

### 2.2. Kemeny Rank Aggregation Approach

The rank aggregation problem may be formulated as follows: given *K* partial rankings of *N* fixed elements, the objective is to find a complete ranking that minimizes the sum of “distances” between itself and each given partial ranking. So, in other words, the ranking aggregation problem is to find a “*consensus*” ranking which reflects the characteristics of given rankings. In particular, the optimal ranking is called *Kemeny optimal rank aggregation* approach [20,21] when the distance is defined as a Kendall tau distance. Genome ranking assigns each genome to a rating vector $\vec{x}$ which most accurately minimizes the sum of tau distances:

(1) $$x^{\tau }=\underset{x}{\text{min}}\left[\overset{K}{\underset{k=1}{\sum }}d_{\tau }\left(\vec{x},r^{k}\right)\right]$$

where *K* is a number of all COGs and where given a rating vector $\vec{x}$ and an “individual” ranking *r^k^* related to COG *k*, *d_τ_* is a Kendall tau distance between them. Kendall tau distance between two permutations is the total number of pairs of elements for which the orders in two permutations disagree.

Informally, the rank aggregation problem is to combine many different rank orderings on the same set of objects in order to obtain the “consensus” ordering. In our case, one may say that every COG proposes its own (partial) ordering of genomes, and finding the function *x*^τ^ (solving Equation (1)) provides the “optimal” ordering. Rank aggregation has been studied in many disciplines, most extensively in the context of social choice theory, where there is a rich literature dating from the latter half of the eighteenth century. By the definition, a Kemeny optimal ranking *x*^τ^ minimizes the total number of pairwise *disagreements* within the sum (1) and maximizes *sortedness*.

Kemeny optimal aggregation has the property of eliminating noise from various different ranking schemes. Furthermore, Kemeny optimal aggregations are essentially the only ones that simultaneously satisfy natural and important properties of rank aggregation functions, called neutrality and consistency in the social choice literature, and the so-called Condorcet property [22]. Indeed, Kemeny optimal aggregations satisfy the *extended Condorcet criterion*.

It is known that finding a Kemeny optimal ranking is NP-hard [23,24]. This motivates the problem of finding a ranking that *approximately* minimizes the number of disagreements with the given input rankings. Given that Kemeny optimal aggregation is useful, but computationally hard, how do we compute it? The sorting procedure, similar to a procedure described in [25], serves as such approximation.

### 2.3. Ranking Methods

There are different methods to measure number of gene copies (we would call these *GFE* measures, which are the *estimates of a level of gene-family extensions*). Genome GFE levels are of interest to us since inter-species variation of genome GFE levels are strongly associated with genome ranking according to number of paralogs. Ranking (or ordering) of objects may be performed in many different ways. Finding an optimal ordering is a nontrivial task. In our review [19] we described several approaches to find a nearly optimal ranking using methods from the field of combinatorial optimization. In this study, we apply four ranking methods: (i) according to an average number (*ave*); (ii) according to a fraction of paralogous gene families (*p.i.*); (iii) according to the sorting procedure (*rank*); and (iv) an index of multi-paralogous families (*mp*).

#### 2.3.1. Average Ranking Method

If *A_i,j_* is the value of *j*th descriptor of the *i*th object, the average ranking method works in this way: for each object *i* the average of all its descriptor values are calculated, which determines the rank of object *i* relative to other objects. All missing values are ignored. In our case, the objects are genomes, the descriptors are COGs and the descriptor values are the quantities of gene copies.

(2) $$ave_{i}=\frac{1}{K^{\prime }}\overset{K}{\underset{j=1}{\sum }}A_{ij}$$

where *K* is a number of all COGs, *A_i,j_* is a number of members in *j*th COG and *i*th genome, and *K′* is a number of gene families in *i*th genome (number of *A_i,j_*’s greater than zero).

#### 2.3.2. Paralog Index

The number of gene-families of size larger than one (non-singletons) divided by the total number of gene-families is called “paralog index” (*p.i.*).

(3) $${p.i.}_{i}=\frac{P}{K^{\prime }}$$

where *P* is an amount of non-singletons, and *K′* is a number of gene families in *i*th genome.

#### 2.3.3. Index of Multi-Paralogous Families

The number of gene-families of size larger than two divided by the number of gene-families with sizes more than one is called “multi-paralog index” (*mp*).

(4) $$mp=\frac{P_{2}}{P}$$

where *P* is an amount of non-singletons, and *P*_2_ is an amount of gene families with more than two copies.

#### 2.3.4. Sort Ranking

We used a procedure similar to a heuristic S-ranking procedure described in [25]. The procedure was applied to an input matrix to rearrange the rows. While we associated a genome with a row in the matrix, the criterion by which adjacent rows (genomes), g1 and g2, were swapped, is as follows: comparing two rows, we considered only gene families present in both genomes, g1 and g2, and counted which row in a pair has larger values more frequently. In other words, if a genome associated with a row *i* has bigger gene-families than a genome associated with a row 𝑖 + 1, then these rows would be swapped. We note that this procedure would not necessarily lead to the optimal ordering. Moreover, the resultant ranking depends on an initial ordering of the objects (genomes). Therefore, we performed 10 runs of the S-ranking procedure starting from randomly chosen orderings and calculated rating vectors $\vec{x}$ (Equation (1)) for each run. After 10 runs, we calculated an averaged rank and its standard deviation for each genome. The standard deviations appeared to be small enough to justify the heuristic S-ranking procedure.

### 2.4. Regression Analysis and Outlier Detection

The relationship between genome sizes and levels of genomic GFE was investigated via the application of correlation and regression analysis. Correlation analysis estimates the statistical significance of the association, whereas regression analysis provides an equation, which precisely describes the relationship. Moreover, this description of the association by equation has predictive value.

In the model selection, two information-based criteria, Bayesian information criterion (BIC) and Akaike information criterion (AIC), were employed to determine the superior model. These criteria balance between goodness of fit and number of parameters in a combined fashion [26]. Minimal scores of AIC determine the best model from a class of models, therefore when fitting a curve to a set of data points, the model with the lowest AIC is chosen. Here, polynomial functions with degrees varying from 1 to 10 were fitted to the data.

A standard method for detecting outliers is boxplot analysis [27]. The notion of a quartile is an essential part of this method. Let us recall the definition of a quartile. Given a sorted list of numbers, the median is a value which divides the data into two parts so that half of numbers are smaller than the median and half are greater than the median. Similarly, quartiles Q1–Q4 split the data into four parts. The second quartile, Q2, is the median [28].

In boxplot analysis the first, second (median) and third quartiles are calculated. From these quantities the interquartile range (IQR), where IQR = Q3 − Q1, is computed, along with two additional values: upper whisker = min(max(*x*), Q3 + 1.5 × IQR) and lower whisker = max(min(*x*), Q1 − 1.5 × IQR). All these quantities are represented in a plot which consists of a box with added “T” shaped lines above and below. The box represents the first and third quartile and the T shaped lines are the upper and lower whiskers. The median is represented as a horizontal line within the box.

Outliers are defined as values outside the range defined by the whiskers. Here, we call these outliers atypical genomes. Once a model is fitted to the data, atypical genomes are determined by applying boxplot analysis on the residuals that is the difference between original (response) and the fitted values. These atypical genomes are marked in the relevant figures as crosses. Analysis was performed with R statistical computing environment [29].

### 2.5. Correlation between GFE Measures

**Figure 1:**
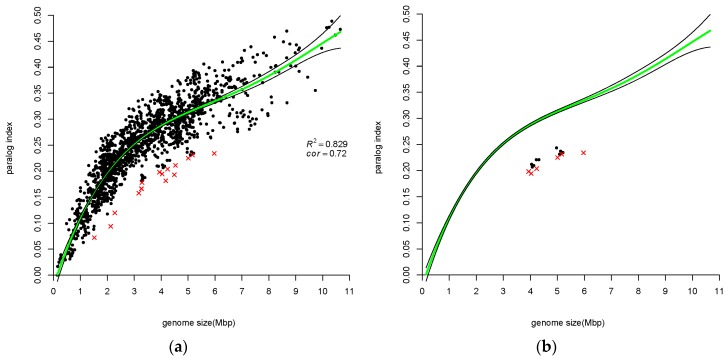
(**a**) Dimension of fraction of paralogous families is plotted versus genome size. Input dataset consists of 1484 prokaryotic genomes. Kendall rank correlation between *p.i.* and genome size is equal to 0.72. Regression polynomial function is 0.25 + 2.69x − 0.71x^2^ + 0.47x^3^ − 0.12x^4^. Regression is found to be statistically significant (F statistic = 1790.059, *p*-value < 2.2 × 10^−16^). Green line shows the fitted model and black lines delimit confidence interval at level of 0.95. Atypical genomes are determined by boxplot analysis on the residuals (see text for details) and are marked by red crosses; (**b**) The same as (**a**) showing only genomes of species from the *Vibrio* genus.

**Figure 2:**
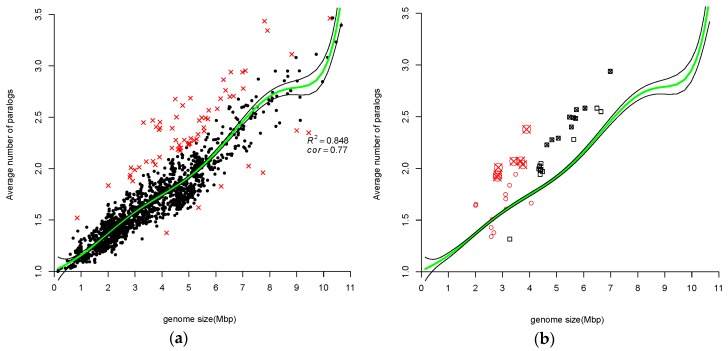
(**a**) Genomic average size of gene-families versus genome size. Kendall rank correlation between average family size and genome size is equal to 0.77. Green line shows the fitted model and black lines delimit confidence interval at level of 0.95. Atypical genomes are determined by boxplot analysis on the residuals (see text for details) and are marked by red crosses. Regression is found to be statistically significant (F statistic = 176.698, *p*-value < 2.2 × 10 ^−16^). Regression polynomial function is 1.66 + 13.92x + 0.82x^2^ + 0.3x^3^ − 0.47x^4^ − 0.02x^5^ + 0.87x^6^ + 0.41x^7^; (**b**) Showing genomes of the species from the Mycobacterium genus (black rectangles and rectangles with crosses mark atypical genomes) and genomes of the species from the Halobacteria class (red circles and circles with crosses mark atypical genomes).

**Figure 3:**
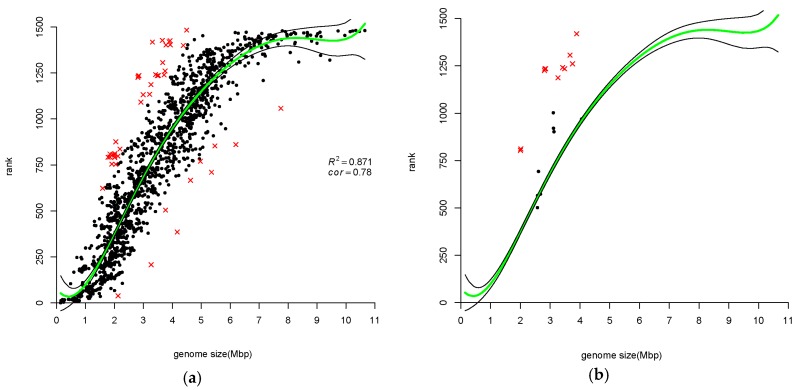
(**a**) Genome ranking versus genome size for the same genomes. Ranking of prokaryotic genomes is performed applying a sorting procedure to the complete input matrix. Kendall rank correlation between a genome rank and its genome size is equal to 0.78. Green line shows the fitted model and black lines delimit confidence interval at level of 0.95. Atypical genomes are determined by boxplot analysis on the residuals (see text for details) and are marked by red crosses. Regression is found to be statistically significant (F statistic = 1672.68, *p*-value < 2.2 × 10^−16^). Regression polynomial function is 741.36 + 14769.57x − 3783.31x^2^ − 641.64x^3^ + 880.83x^4^ − 344.26x^5^ + 277.53x^6^; (**b**) Shows (magnifies) the genomes of the species from the Halobacteria class.

**Figure 4:**
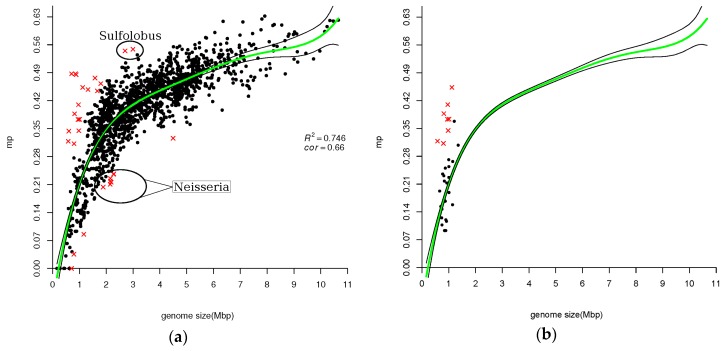
Relative frequency of larger gene families *mp* = <number of gene-families with more than two gene copies>/<total number of non-singletons> (**a**) Relationship between *mp* index versus genome size in the same prokaryotic genomes. Kendall rank correlation between *mp* and genome size is equal to 0.66. Green line shows the fitted model and black lines delimit confidence interval at level of 0.95. Atypical genomes are determined by boxplot analysis on the residuals (see text for details) and are marked by red crosses. Regression is found to be statistically significant (F statistic = 722.90, *p*-value < 2.2 × 10^−16^). Regression polynomial function is 0.4 + 3.13x − 1.17x^2^ + 0.81x^3^ − 0.44x^4^ + 0.23x^5^ − 0.01x^6^; (**b**) Relationship between *mp* index versus genome size in Mycoplasmas.

When a set of variables are related, estimating the correlation between a pair of variables using standard methods, e.g., Kendall’s tau, is uninformative since standard correlation methods ignore the knowledge that the specific pair of variables are correlated with other variables. Partial and semi-partial correlation methods are modifications of the standard methods, which take into account correlations to other variables. Partial correlation is used when a pair of variables, say *x* and *y*, are both correlated with a variable *z*. The coefficient expresses the residual correlation between variables *x* and *y* after eliminating the correlations between variables *x* and *y* with variable *z*. Figure 1, Figure 2, Figure 3 and Figure 4 show that all measures of paralog indices are correlated with genome-size.

Therefore, we estimated the correlation between these indices by calculating Kendall’s tau (partial) correlation coefficient using ppcor R package [30].

## 3. Results

### 3.1. Number of Paralogs is Correlated with Genome Size

Here we examine associations between gene-family size and genome size using different measures of number of paralogs in order to analyze the universality of the trend and to highlight factors possibly influencing deviations from the common trend. In addition, in some cases we examine associations between gene-family size and number of genes.

#### 3.1.1. Percentage of Paralogous Gene Families is Correlated with Genome Size

We divided all protein-coding genes into two categories: singletons and appearing in more than one copy, i.e., belonging to paralogous families. The number of paralogous families divided by the total number of gene families is called “paralog index” (*p.i.*). Pushker et al. [4] applied a closely related measure to 127 eubacterial genomes. (Pushker et al. used the following definitions: *p.i.* is a percentage of paralogs in the genome (genes with at least one local BLAST hit using the cut-offs) among all genes; *ave* is an average size of paralogous families (singletons are excluded).) Here, we applied *p.i.* to 1484 prokaryotic genomes and show the results in Figure 1a, where paralog index is plotted vs. genome sizes. Correlation between paralog index and genome size is clearly seen and the values of correlation coefficients are as follows: Spearman correlation is equal to 0.896, Pearson correlation is equal to 0.866 and Kendall rank correlation is equal to 0.723. We considered the latter correlation coefficient as the most relevant when analyzing ranking results; therefore, it was chosen for herein analysis. Actually, we see that the association of paralog index with genome size is different for small genomes as compared with larger genomes. A “break point” is located somewhere around 2.2 Mbp. The linear regression equation for small genomes is approximately y ≈ 0.1x, while for larger genomes it is y ≈ 0.03x + 0.15. The paralog index for smaller genomes grows faster with an increase of genome size compared to larger genomes. We can see that the data follow different linear trends over different regions of the data, so one can use piecewise linear regression, modeling the regression function in “pieces”. We preferred to apply the polynomial regression approach to all four measures of “genome GFE”.

The presented polynomial regression lines were chosen based on AIC criterion (see Materials and Methods). The regression polynomial function is 0.25 + 2.69x − 0.71x^2^ + 0.47x^3^ − 0.12x^4^. There are outliers among both small and larger genomes. Interestingly, all outliers related to *p.i.* are located under the regression line, which means that outliers have a **smaller** fraction of paralogous gene-families than would be predicted by regression analysis. There are 16 outliers including *M. leprae* and 6 Vibrio genomes (see Table 1).

There are 15 Vibrio genomes in our dataset. They are shown in Figure 1b. We can see that they all make a cluster, while 6 Vibrio genomes are outliers and 9 genomes are “almost” outliers.

#### 3.1.2. Average Number of Paralogs Correlate with Genome Size

In Figure 2, average size of a gene family (Equation (2)) in a given genome is plotted vs. the size of that genome. Correlation between average number of all gene copies in all COGs and genome size is clearly seen with the Kendall rank correlation equal to 0.767. Interestingly, unlike in Figure 1, here in Figure 2 we observe similar behavior between small and larger genomes. Ranking of objects based on average value across all nonzero attributes is known to be an oversimplified ranking method. Figure 2 is very noisy, indeed. If for *p.i.* only 16 genomes were detected as outliers, which is about 1% of all the examined genomes, for *ave* 67 genomes were detected as outliers, which is a larger fraction of the analyzed genomes (~4.5%). Thus, only a partial list of the outliers is shown in Table 2. (The complete list of the *ave* outliers is in the Supplementary materials Table S1.) There are individual representatives of different taxa among these outliers, including Pirellula, Bordetella, Burkholderia, etc.; however, we decided to show in Figure 2b only two highly represented groups, Mycobacterium genus and Halobacteria class (see also, Table S1).

Some genomes of Mycobacterium genus and Halobacteria have smaller average gene-family sizes than would be predicted by the regression polynomial function but, interestingly, all outliers of these two groups appear above the regression line (Figure 2b). From Table 2 we can make another interesting observation: all four Rhodococcus genomes are among the outliers. We hypothesize that an explanation of an incidence of Rhodococcus occurring in the group of outliers would be the same as for Mycobacteria, because Rhodococcus genus is closely related to Mycobacterium genus.

#### 3.1.3. Ranking of Prokaryotic Genomes Based on Gene-Family Size Confirms Correlation with Genome Size

As we described in Materials and Methods, we used a sorting procedure to rank genomes according to their family sizes. In Figure 3, genome rank is plotted vs. size of that genome. This ranking method results in genome ordering close to Kemeny optimal [31]. Correlation between average number of all gene copies in all COGs and genome size is clearly seen for Kendall rank correlation (0.78). There are 46 outliers of the regression model constructed for rank measure. They are placed in Supplementary Materials Table S2. Twenty-four out of these 46 outliers belong to the Archaea kingdom; half of these 24 Archaea belong to Halobacteria class and 5 of the remaining 12 Archaea are from Crenarchaeota. A partial list of the outliers is shown in Table 3.

We recognized some genomes of Mycobacterium genus as outliers of the regression model constructed for *ave* measure. None of them appear in Table S2. However, *Mycobacterium leprae*, which was not among outliers presented in Table S1, appears in Table 1 and Table S2. Halobacteria were among *ave* measure outliers in Table S1, and there are 12 Halobacteria in Table S2 as well. We show Halobacteria data in Figure 3b.

### 3.2. Fraction of Larger Gene-Families

In parallel to a paralog index (Figure 1), we calculated another simple measure of GFE. It is relative frequency of larger gene families:

$$mp=\frac{\text{number of gene families with more than two gene copies}}{\text{total number of non}-\text{singletons}}$$

In Figure 4, *mp* fraction is plotted vs. genome size. Interestingly, there is a striking shape-similarity between Figure 1 and Figure 4. In Figure 4, we see that association of *mp* with genome size is different for small genomes as compared with larger genomes (like it was for paralog index—see Figure 1). In the case of *mp*, a “break point” is located somewhere around 2.3 Mbp, similar to *p.i.* Small genomes produce a smear cloud of points with multiple outliers, while for larger genomes a linear regression line y ≈ 0.02x + 0.32. The regression polynomial function is 0.4 + 3.13x − 1.17x^2^ + 0.81x^3^ − 0.44x^4^ + 0.23x^5^ − 0.01x^6^. There are outliers among both small and larger genomes but mainly among the smaller ones. Among larger genomes there are a few genomes of Neisseria and Sulfolobus. Neisseria outliers have a **smaller** fraction of multiple paralogous gene families than would be predicted by regression analysis, while Sulfolobus show the opposite effect. Altogether, there are 29 outliers including 6 Phytoplasmas and 8 Mycoplasmas (see Table 4 and Table S4). Mycoplasmas are shown in Figure 4b. It seems that there is no correlation between genome size and *mp* for Mycoplasmas. For some of them, *mp* indices may be predicted pretty well by the regression polynomial function, and some of them are outliers. The latter are listed in Table 4.

## 4. Discussion

### 4.1. Number of Gene Copies Is Correlated with Genome Size

Correlation between gene-family size measured by paralog index and number of genes was discovered many years ago [2]. Huynen and van Nimwegen showed that an increase in the number of genes leads not only to an increase in the number of gene copies, but also to a relative increase of the number of large gene families over the number of small families. They obtained these results comparing complete genomes of six bacteria (*E. coli*, *H. influenzae*, *H. pylori*, *M. genitalium*, *M. pneumoniae*, and *Synechocystis* sp. PCC6803) and two Archaea (*M. jannaschii* and *M. thermoautotrophicum*). Huynen and van Nimwegen wrote [2] “as more genomes become available; it will be possible to analyze how general the observed trend is”.

In early 2000s, the following rule was stated several times on growing number of sequenced prokaryotic genomes: The number of paralogous genes and families are positively correlated with an increase in genome size [3,4,11,15,16]. Pushker et al. stated that “the relative contribution of paralogous genes in each genome seems to be independent of phylogenetic affiliation and, for a limited dataset, appears to depend on genome size” [4].

Our calculations, performed on much larger dataset, confirmed the above-mentioned rules, in general. In all mentioned above publications from 2000s, only the simplest ranking methods were applied to the problem. We decided to apply Kemeny optimal aggregation, which is one of the most adequate ranking methods [20,21]. This method produced ordering of genomes different from the simpler methods; however, all measures highly correlate. The correlation levels are moderate, yet highly significant (*p*-values < 2.2 × 10^−16^), therefore it is likely that these different measures highlight the same underlying core phenomenon. This phenomenon is so strong that even the averaging method, often giving untruthful results, is rather comparable with the valid Kemeny method, in this case. Regarding atypical genomes, which are method-dependent ones, we propose to put more trust into the results produced by the latter technique (Figure 3, Table 3).

### 4.2. Atypical Genomes

We detected some genomes as outliers via the application of a boxplot analysis. We referred to these genomes as atypical in a sense that they are “far” from the trend found in Figure 1, Figure 2, Figure 3 and Figure 4. They were marked by red crosses and are listed in their respective complete and partial lists of atypical genomes (Table 1, Table 2 and Table 3, Table 5, Table 7, Table S1, Table S4). Notably, certain taxa are omnipresent or, in other words, they are atypical with respect to all three measures of GFE (e.g., *Candidatus* Cloacamonas acidaminovorans Evry, Pirellula and Orientia). Other taxa are almost omnipresent (e.g., *Mycobacteriaceae* family, *Halobacteria* class). The Mycoplasmas are the predominant family with regard to *mp* index (Table 4). Likewise, genomes of the Neisseria family are atypical, also with respect to *mp* index. Taxonomy statistics of outliers (i.e., species combined in taxa with the corresponding number of species within each taxon) were calculated (see Table S4).

Let us compare our outliers with the outliers found by our predecessors. Huynen and van Nimwegen [2] found an outlier studying a rather small sample of eight prokaryotes: *M. pneumoniae*, showed a relatively high frequency of large gene families. Pushker et al. [4] identified several genomes with atypical *mp* values: *Mycoplasma pneumoniae*, *Mycoplasma penetrans*, and *Mycoplasma gallisepticum*. Our results also show that Mycoplasmataceae is worth a separate discussion, which is below. Pushker et al. [4] also mentioned the following outliers: *Mycobacterium leprae*, *Pirellula* sp., *Shigella flexneri*, *Bordetella pertusis*, *B. parapertussis,* and *B. bronchiseptica*. Our results only partly confirmed these observations. *M. leprae* is discussed below in a separate subsection devoted to Mycobacteriaceae family. Likewise, a separate subsection is devoted to Pirellula. *Shigella flexneri* is not an outlier (Tables S3 and S4). Yet two members of the Bordetella species were found as outliers for the average number of gene copies, *B. bronchiseptica RB50* and *B. petrii* (Table S1).

#### 4.2.1. Mycoplasmas

In Table 5, we show gene-family sizes of Mycoplasmataceae. In column titled 1, we present number of singletons, in columns 2 and 3, amounts of gene-families of two and three copies, correspondingly. Mycoplasmas have small genomes with amounts of COG-annotated proteins (NC) varying from ~250 to 700 proteins. Fraction of singletons “1”/NC is more or less invariant at about 70%–80%. *mp* measures relative frequency of gene-families with more than two copies per family: *mp* = <number of gene-families with more than two gene copies>/<total number of non-singletons>. For *Mycoplasma fermentans* M64, for example, *mp* is equal to 0.45, while an expected value is about 0.26. There are 383 singletons, 35 gene families composed of two copies each, 11 gene families of 3 gene copies, and 18 families with more than three gene copies. *mp* = (29 = 11 + 18)/(64 = 35 + 11 + 18). Total number of non-singletons is equal to 64 and this is expected number of paralog families (*M. fermentans* is not an outlier for the measures *p.i.*, *ave* and *rank*), while 29 is a surprisingly high number of gene-families with more than two gene copies. We do not have an answer to the question “Why *M. hyopneumoniae* has a low *mp* index while *M. bovis* Hubei has a high one” (study in progress).

Pushker et al. [4] estimated *Mycoplasma gallisepticum* as an atypical genome according to an average number of gene copies but it is not in our list of outliers (Table S1). Our calculations of *ave* show that *M. gallisepticum* has an average number of gene copies equal to 1.2, which is close to an expected value. Probably, differences both in calculations of an average number of gene copies and of outliers result in dissimilar outcomes. Pushker et al. [4] also identified two Mycoplasmas with atypical *mp* values: *Mycoplasma pneumoniae* and *Mycoplasma penetrans*. These two genomes appear in Table 4 as well.

#### 4.2.2. Mycobacterium

General considerations suggested that large genetic diversity should exist among *M. leprae* strains, however, comparative genomics revealed that genetic variation was found to be exceptionally rare [32,33]. All indices for two strains of *M. leprae* are practically identical, so, we would use a term “species” instead of discussing the two genomes separately. *M. leprae* is an outlier in two categories: *p.i.* is equal to 0.17, while the expected value is about 0.25; *rank* is equal to 207, while the expected value is about 740; *ave* is equal to 1.32, which is not so close to expected 1.55, but it is only an “almost outlier”. Interestingly, in the two categories in which *M. leprae* is an outlier, all other members of this genus are absent. In the category *ave*, 10 non-tuberculosis Mycobacteria are outliers (Figure 2b) but *ave* is the noisiest and less reliable index of GFE; thus we would consider only *M. leprae* as a paralog-atypical species. In the context of mycobacterial species, Mycobacterium leprae has the smallest genome as a result of massive reductive evolution. The differences in the total number of protein-coding genes and number having homolog genes between *M. leprae* and all other Mycobacteria are striking (Table S5). Actually, all Mycobacteria but *M. leprae* have rather similar genomic characters. There were several attempts to explain this well-known observation (see [34] and references therein), but still the very special reduced evolution of *M. leprae* requires additional studies to give a plausible explanation. Despite over a century of research we still lack a clear understanding of the pathogenesis and physiology of this pathogen. Even basic epidemiologic and genomic questions are yet to be resolved completely. Reasonable speculation would say that reductive evolution results in low level of paralogization; but evolution has worked on *M. leprae* by controversial means: low number of gene copies from one side and having the largest proportion of pseudogenes in comparison to other prokaryotes from the other side [32]. About 50% of the *M. leprae* genome is seemingly devoid of function [32,35]. Comparative genomics of *M. leprae* is a challenging task.

#### 4.2.3. Halophiles

Sanchez-Perez et al. [36] proposed a very reasonable hypothesis of environmental adaptation. The idea is that the original and paralog (i.e., copy) gene share the same function, yet, the paralog gene is expressed under abnormal environmental conditions (They named these kinds of paralogs ecoparalogs). One example is the hyperhalophilic bacterium Salinibacter ruber. This bacterium has halophilic proteins that have their optimal activity and stability at high salinity. Sanchez-Perez et al. also found examples of ecoparalogs in other prokaryotes. We are investigating whether ecoparalogization is the main reason for majority of Halophiles having enlarged gene families (work in progress). Comparative genomics is the right instrument for this kind of analysis.

#### 4.2.4. Pirellula

A marine bacterium Pirellula appears as an outlier both for *ave* and *S-rank* measures (Tables S1 and S2). We are not the first to recognize this species as an outlier. Already Pushker et al. have mentioned, “Pirellula has an enormous genome with a surprisingly low relative number of paralogs” [4]. An appearance of Pirellula in Tables S1 and S2 and absence from Table 1 is due to an overrepresentation of small gene families and the absence of large ones. Pirellula is a marine bacterium and Pushker et al. suggested that the reason for the reduced gene-family size might be the homogeneity of the marine environment. For instance, Pirellula has a greatly reduced number of transcriptional regulators [37]. There are four genomes even bigger than Pirellula with “a surprisingly low relative number of paralogs”. *Trichodesmium*, also called sea sawdust, are found in tropical and subtropical ocean waters. *Hahella chejuensis* is a marine microbe. *Haliangium ochraceum* is a species of moderately halophilic Myxobacteria. *Myxococcus fulvus* is a species from the *Myxococcaceae* family. From these five genomes (Table 6) Pirellula and Trichodesmium are rank-outliers and, as such, appear in Table S2 as well. Both are marine bacteria.

The idea that “Gene duplications in prokaryotes can be associated with environmental adaptation” [38] looks very reasonable. In Halophiles, environmental adaptation results in **expanded** gene-families, while in big marine bacteria it results in **reduced** gene-family size.

#### 4.2.5. *Orientia tsutsugamushi*

*Orientia tsutsugamushi* (OT), an obligate intracellular bacterium belonging to the family Rickettsiaceae of the subdivision alpha-Proteobacteria, is the causative agent of scrub typhus, or Tsutsugamushi disease. The complete genome sequences of two OT strains were obtained and COG-annotated [39,40]. Both strains have a single circular chromosome and possess no plasmid. The chromosomes are very similar in size (2,008,987 bp in Ikeda and 2,127,051 bp in Boryong) with almost identical average G + C contents (30.5% in both strains). The numbers of rRNA and tRNA genes are identical. The numbers of protein-coding genes and pseudogenes, the coding content, and the repeat content were identified by Nakayama et al. [41].

OT appears as an outlier in all three paralog measures. *Orientia tsutsugamushi* Ikeda has a surprisingly **high** average number of gene copies (1.83 instead of expected 1.36). *Orientia tsutsugamushi* Boryong has a surprisingly **low** paralog index (0.09 instead of expected 0.2) and **low** rank (38 vs. 480) (Table S6, Figure 1, Figure 2 and Figure 3). Genomic analysis of the two OT strains revealed that extensive reductive genome evolution as well as explosive and comprehensive amplification of repetitive sequences have occurred in OT. In both strains, repetitive sequences occupy nearly half the genome [40,41].

Nakayama et al. [40,41] defined OT paralogs as the genes whose products exhibited at least 90% amino acid sequence identity over 60% of the alignment length. According to this definition, they found 1196 repeated genes that were classified into 85 OT paralogous gene families. Extensive gene decay has taken place in many Boryong-repeated genes as in those of Ikeda. We used a rather different gene copy definition and our results are 772 paralogous genes that were classified into 115 OT paralogous gene families.

Analyzing Table S6 we can conclude that all parameters excluding genome size are pretty similar among all Rickettsiaceae. Our hypothesis regarding OT being an outlier is that in the case of OT, genome size is not a relevant genomic characteristic because of very large number of repetitive sequences.

### 4.3. Ranking Methods

The objective of the study was to find associations between characteristics of genomic gene family sizes and other genomic attributes, like genome size. We believe that the ranking of genomes according to a gene family size, followed by the calculation of coefficients of association between genome rank and genome property, is a reasonable approach in revealing hidden driving factors. The goal is to rank genomes in a way such that genomes with lower number of gene copies would have lower rank. In this study we used different methods to rank genomes (see Methods): according to (i) an average number (*ave*); (ii) a fraction of paralogous gene families size (*p.i.*); (iii) the sorting procedure (*rank*); and (iv) a fraction of multi-paralogous families (*mp*).

In order to compare different methods of ranking, Kendall tau rank correlation coefficients were calculated. Since all measurements of “GFE levels” of a genome are correlated with a genome size (Figure 1, Figure 2, Figure 3 and Figure 4), the partial correlation was calculated (that is controlling for effects of genome size on the estimated correlations). The coefficients are shown in Table 7.

**Should** these three indexes **inevitably** correlate? Not necessarily. In Table 8 we show an example of imaginary data to illustrate different estimates of the levels of paralogization.

Genome sizes sort out the genomes in the order of B, A, C; *p.i.* – A, C, B; *ave* – B, C, A; *rank* – A, B, C; *mp* – B, C, A. *p.i.*, *ave* and *rank* characterize differently the distribution of gene-family sizes in the three genomes A, B, C. In our fictitious example none of the indices gives the order B, A, C, the order of genome sizes. We would not say that only one of the indices is correct but, instead, we propose to consider all three estimates of GFE. Each estimate produces its own set of outliers, which we discussed above, and only several genomes belong to intersection of outliers’ sets: *Candidatus* Cloacamonas acidaminovorans Evry, *Pirellula* and *Orientia* are omnipresent; *Mycobacterium leprae* and many *Halobacteria* appear in two subsets.

## 5. Conclusions

In earlier works it was found that number of paralogs and size of genome are positively correlated. This result was achieved using the simplest methods of estimation of genomic number of paralogs. In this study, we reexamined these associations on a larger dataset consisting of 1484 prokaryotic genomes and using several ranking approaches including the Kemeny optimal aggregation approach. We found that for all measures of GFE associations between a measure and a genome size follow different approximately linear trends over different genome sizes. Until now, only linear regression models were applied to the model of gene-family size–genome size association. We preferred to apply the polynomial regression approach to all four measures of “genome GFE”. The polynomial regression lines were chosen based on AIC criterion. For more rigorous description, boxplot analysis was used for outlier detection.

We confirmed that number of gene copies positively correlates with an increase of genome size. As expected, different groups of atypical prokaryotic genomes were found for different types of gene-family-size quantities. We confirmed that *M. leprae* has a substantially lower number of gene copies than would be expected from its genome size. We found that the majority of the members of Mycoplasmataceae possess a surprisingly high number of gene-families with more than two gene copies. We obtained sound reasoning for the speculation that in Halophiles, environmental adaptation results in expanded gene families, while in big marine bacteria it results in the reduced gene family size.

All the above-mentioned results were obtained by applying different measures of genomic number of gene copies. We propose to use all four estimates of GFE because they may mirror different aspects of GFE. Kendall tau partial rank correlation coefficients were calculated between different measurements of “GFE levels”. They are all pairwise correlated and separately correlate with genome size, and all these correlations were found to be statically significant.

In summary: we not only demonstrated that previously found associations between genome size and characteristics of gene-families were corroborated on a considerably larger dataset of prokaryotic genomes; we also utilized additional ranking methods for more accurate descriptions of these associations and highlighted atypical microbes and whole taxonomic groups. Our results show that examination of gene-duplication history in these taxa may provide especially valuable insights into the underlying evolutionary processes.

## Figures and Tables

**Table 1:** Atypical genomes according to a paralog index measure ^1^.

*Rank*	*p.i.*	Size (Mb)	Atypical Genomes
34.8	0.072	1.516	*Ehrlichia ruminantium* Welgevonden
38.3	0.094	2.127	*Orientia tsutsugamushi* Boryong
106	0.120	2.279	*Treponema pallidum* SS14
379	0.158	3.168	*Prevotella melaninogenica* ATCC25845
207	0.166	3.268	*Mycobacterium leprae* Br4923
208	0.166	3.268	*Mycobacterium leprae* TN
611	0.178	3.286	*Brucella abortus* bv 19941
769	0.198	3.939	*Vibrio cholera* M662
763	0.194	4.033	*Vibrio cholera* O1 biovar ElTor N16961
385	0.182	4.171	*Sodalis glossinidius morsitans*
820	0.204	4.236	*Vibrio cholera* MJ1236
1483	0.193	4.494	*Candidatus* Cloacamonas acidaminovorans Evry
787	0.211	4.532	*Aliivibrio salmonicida* LFI1238
1072	0.225	5.008	*Vibrio vulnificus* MO624O
1281	0.231	5.166	*Vibrio parahaemolyticus* RIMD2210633
1293	0.234	5.969	*Vibrio harveyi* ATCCBAA1116

^1^ *p.i.*—paralog index, *Rank*—is an averaged rank calculated for multiple runs of the S-ranking procedure. Genomes are sorted by ascending size of genome for easier comparison with Figure 1.

**Table 2:** Partial list of atypical genomes according to average number of paralogs ^1^.

*Rank*	*Ave*	Size (Mb)	Atypical Genomes
246.8	1.521	0.853	*Onion yellows phytoplasma* OYM uid58015
			…
1225.1	1.915	2.809	*Halalkalicoccus jeotgali* B3 uid50305
1233.4	1.936	2.821	*Halogeometricum borinquense* DSM
1235.3	2.008	2.848	*Haloferax volcanii* DS2 uid46845
1091.1	1.878	2.914	*Halophilic archaeon* DL31 uid72619
1240.8	2.067	3.420	*Haloarcula marismortui* ATCC 43049 uid57719
1306.5	2.071	3.668	*Halopiger xanaduensis* SH6 uid68105
1260.9	2.036	3.752	*Natrialba magadii* ATCC 43099 uid46245
1419.5	2.378	3.889	*Haloterrigena turkmenica* DSM 5511
			…
948.4	2.228	4.644	*Mycobacterium* JDM601 uid67369
1074.7	2.277	4.830	*Mycobacterium aviumparatuberculosis* K10
1211.8	2.293	5.067	*Mycobacterium abscessus* uid61613
1074.9	2.495	5.475	*Mycobacterium avium* 104 uid57693
1275.8	2.399	5.548	*Mycobacterium gilvum* Spyr1 uid61403
1303.6	2.491	5.620	*Mycobacterium gilvum* PYRGCK uid59421
1306.9	2.483	5.705	*Mycobacterium* MCS uid58465
1320.9	2.567	5.737	*Mycobacterium* KMS uid58491
1319.4	2.582	6.048	*Mycobacterium* JLS uid58489
1449.2	2.938	6.988	*Mycobacterium smegmatis* MC2155 uid57701
			…
1477.8	3.463	10.237	*Amycolatopsis mediterranei* U32 uid50565

^1^ *Rank*—is an averaged rank calculated for multiple runs of the S-ranking procedure; *ave*—average number of paralogs.

**Table 3:** Partial list of atypical genomes according to *S-Rank*.

*Rank*	Size (Mb)	Atypical Genomes
622.8	1.591	*Candidatus* Korarchaeum cryptofilum OPF8
		…
803.4	2.001	*Halobacterium salinarum* R1
811.5	2.014	*Halobacterium* NRC1
1225.1	2.809	*Halalkalicoccus jeotgali* B3
1233.4	2.821	*Halogeometricum borinquense* DSM11551
1235.3	2.848	*Haloferax volcanii* DS2
1091.1	2.914	Halophilic archaeon DL31
1186.8	3.261	*Halorubrumlacus profundi* ATCC 49239
1240.8	3.420	*Haloarcula marismortui* ATCC 43049
1235.0	3.484	*Haloarcula hispanica* ATCC 33960
1306.5	3.668	*Halopiger xanaduensis* SH6
1260.9	3.752	*Natrialba magadii* ATCC 43099
1419.5	3.889	*Haloterrigena turkmenica* DSM 5511
		…
1057.6	7.750	*Trichodesmium erythraeum* IMS101

**Table 4:** List of atypical genomes according to *mp* ^1^.

*Rank*	*mp*	Size (Mb)	Atypical Genomes
31.2	0.32	0.580	*Mycoplasma genitalium* G37
166.6	0.34	0.602	*Candidatus* Phytoplasma Mali
21.9	0.00	0.706	*Candidatus* Blochmannia floridanus
246.7	0.49	0.707	Aster yellows witches broom phytoplasma AYWB
11.5	0.04	0.792	*Candidatus* Blochmannia pennsylvanicus BPEN
183.6	0.31	0.799	*Mycoplasma synoviae* 53
31.8	0.39	0.816	*Mycoplasma pneumoniae* M129
246.8	0.49	0.853	Onion yellows phytoplasma OY M
167.2	0.48	0.880	*Candidatus* Phytoplasma australiense
192.8	0.37	0.948	*Mycoplasma bovis* Hubei 1
199.5	0.41	0.964	*Mycoplasma pulmonis* UAB CTIP
191.8	0.34	0.978	*Mycoplasma fermentans* JER
297.6	0.37	1.007	*Mycoplasma agalactiae*
186.2	0.45	1.119	*Mycoplasma fermentans* M64
77.1	0.09	1.161	*Candidatus* Ruthia magnifica Cm *Calyptogena magnifica*
420.9	0.45	1.317	*Thermosphaera aggregans* DSM 11486
411.0	0.48	1.580	*Staphylothermus hellenicus* DSM 12710
358.7	0.44	1.667	*Gardnerella vaginalis* ATCC 14019
481.4	0.46	1.796	*Streptococcus thermophilus* CNRZ1066
196.2	0.20	1.887	*Haemophilus influenzae* PittGG
156.9	0.21	2.145	*Neisseria meningitidis* alpha14
158.0	0.22	2.153	*Neisseria meningitidis* 053442
154.3	0.23	2.154	*Neisseria gonorrhoeae* FA 1090
160.0	0.22	2.184	*Neisseria meningitidis* Z2491
166.2	0.24	2.272	*Neisseria meningitidis* MC58
105.6	0.24	2.279	*Treponema pallidum* SS14
859.3	0.54	2.702	*Sulfolobus islandicus* Y G 57 14
1131.5	0.55	2.992	*Sulfolobus solfataricus* P2
1483.0	0.33	4.494	*Candidatus* Cloacamonas acidaminovorans Evry

^1^ *Mp* = <number of gene-families with more than two gene copies>/<total number of non-singletons>.

**Table 5:** Distribution of gene-family sizes of Mycoplasmataceae ^1^.

Genome Name	Np	NO	NC	1	2	3	>3	*mp*
*M. agalactiae* PG2	742	267	475	335	42	10	4	14/56
*M. agalactiae* uid46679	813	291	522	332	42	15	10	25/67
*M. arthritidis* 158L3 1	631	214	417	347	20	3	3	6/26
*M. bovis* Hubei 1	801	279	522	346	37	11	11	22/59
*M. bovis* PG45	765	239	526	354	43	9	7	16/59
*M. capricolum* ATCC 27343	812	236	576	390	58	10	7	17/65
*M. conjunctivae*	692	272	420	323	39	0	4	4/43
*M. crocodyli* MP145	689	199	490	380	37	6	4	10/47
*M. fermentans* JER	797	247	550	388	38	8	12	20/58
*M. fermentans* M64	1049	459	590	383	35	11	18	29/64
*M. gallisepticum* R low	763	274	489	357	43	4	6	10/53
*M. genitalium* G37	475	91	384	330	15	4	3	7/22
*M. haemofelis* Langford 1	1545	1258	287	230	16	2	1	3/19
*M. hominis* ATCC 23114	523	145	378	315	21	1	4	5/26
*M. hyopneumoniae* 232	691	254	437	331	39	1	3	4/43
*M. hyopneumoniae* 7448	657	214	443	333	38	1	4	5/43
*M. hyopneumoniae* J	657	186	471	344	44	2	4	6/50
*M. hyorhinis* HUB 1	658	194	464	339	36	7	2	9/45
*M. leachii* PG50	882	316	566	398	50	9	8	17/67
*M. mobile* 163K	633	183	450	370	26	6	2	8/34
*M. mycoides capri* LC 95010	922	303	619	400	55	6	14	20/75
*M. mycoides* SC PG1	1017	325	692	397	55	15	16	31/86
*M. penetrans* HF 2	1037	379	658	447	54	10	14	30/84
*M. pneumoniae* M129	648	203	445	359	19	6	6	12/31
*M. pulmonis* UAB CTIP	782	222	560	387	36	8	17	25/61
*M. putrefaciens* KS1	650	176	474	379	34	4	3	7/41
*M. suis* Illinois	845	592	253	209	14	0	2	2/16
*M. suis* KI3806	794	553	241	212	11	1	1	2/13
*M. synoviae* 53	659	180	479	357	33	10	5	15/48
*U. parvum serovar* 3 ATCC 27815	609	196	413	346	25	1	2	3/28
*U. parvum serovar* 3 ATCC 700970	614	173	441	360	29	3	2	5/34
*U. urealyticum serovar* 10 ATCC 33699	646	230	416	342	25	3	2	5/30

^1^ NP—number of proteins; NO—number of ORFans; NC—number of COG-annotated proteins; *M.*—Mycoplasma; *U.*—Ureaplasma.

**Table 6:** Partial list of atypical genomes according to average number of gene copies.

*Rank*	*Ave*	Size (Mb)	Atypical Genomes
861	1.827	6.196	*Pirellula staleyi* DSM_6068_uid43209
1341	2.024	7.215	*Hahella chejuensis* KCTC_2396_uid58483
1058	1.961	7.750	*Trichodesmium erythraeum* IMS101_uid57925
1411	2.370	9.004	*Myxococcus fulvus* HW_1_uid68443
1319	2.349	9.446	*Haliangium ochraceum* DSM_14365_uid41425

**Table 7:** Pairwise partial Kendall correlation between all ranking methods ^1^.

	*p.i.*	*Ave*	*Rank*	*mp*	Genome Size
*p.i.*		0.57	0.57	0.46	0.72
*ave*	0.57		0.61	0.52	0.77
*rank*	0.57	0.61		0.38	0.78
*mp*	0.46	0.52	0.38		0.66

^1^ All correlations were controlled for genome size and are statistically significant (*p*-value < 2.2 × 10^−16^).

**Table 8:** The indices of GFE of fictional data.

Genome	ORFans	COGs					*p.i.*	*Ave*	*Rank*	*Mp*
		1	2	3	4	5				
**A**	10	1	1	1	1	16	0.2	4	1	1.0
**B**	8	1	1	2	2	4	0.6	2	2	0.3
**C**	20	1	1	1	6	6	0.4	3	3	0.7

## Supplementary Materials

The following are available online at http://www.mdpi.com/2075-1729/6/3/30/s1: Complete table of results, Table S1: Complete list of atypical genomes according to average number of gene copies, Table S2: Complete list of atypical genomes according to *S-Rank*, Table S3: GFE indices of Shigellas, Table S4: Taxonomy of outliers, Table S5: Distribution of gene-family sizes of *Mycobacteriaceae*, Table S6: *Orientia tsutsugamushi* and *Rickettsia*.

## Abbreviations

The following abbreviations are used in this manuscript:

COG	Cluster of Orthologous Groups of proteins
HGT	Horizontal Gene Transfer
GFE	gene-family extension
Mbp	millions of base pairs
OT	*Orientia tsutsugamushi*
*p.i.*	the number of protein-coding gene families having more than one copy divided by the total number of COG-annotated protein-coding gene families
*ave*	average size of protein-coding gene families, including singletons
*mp*	the number of protein-coding gene families having more than two copies divided by the number of protein-coding gene families having more than one copy
