# Supplementary material for: Gene-Family Extension Measures and Correlations
Source: Life (Basel). 2016 Aug 3;6(3):30. doi: 10.3390/life6030030 (PMC5041006; doi:10.3390/life6030030)
Supplement: Supplementary file 1 [file life-06-00030-s001.pdf]

Supplementary Materials: Gene-Family Extension Measures and Correlations

Gon Carmi and Alexander Bolshoy

Table S1. Complete list of atypical genomes according to average number of paralogs [1].

Rank	Ave	Size (Mb)	Atypical Genomes
246.8	1.521	0.853	Onion yellows phytoplasma OY M uid58015
66.8	1.835	2.009	*Orientia tsutsugamushi* Ikeda uid58869
1225.1	1.915	2.809	*Halalkalicoccus jeotgali* B3 uid50305
1233.4	1.936	2.821	*Halogeometricum borinquense* DSM
1235.3	2.008	2.848	*Haloferax volcanii* DS2 uid46845
1091.1	1.878	2.914	*Halophilic archaeon* DL31 uid72619
1131.5	1.987	2.992	*Sulfolobus solfataricus* P2 uid57721
1073.9	2.012	3.155	*Renibacterium salmoninarum* ATCC 33209
1417.6	2.447	3.311	Azospirillum B510 uid46085
1240.8	2.067	3.420	*Haloarcula marismortui* ATCC 43049 uid57719
1426.9	2.469	3.654	*Sinorhizobium meliloti* 1021 uid57603
1306.5	2.071	3.668	*Halopiger xanaduensis* SH 6 uid68105
1260.9	2.036	3.752	*Natrialba magadii* ATCC 43099 uid46245
1401.9	2.407	3.782	*Sinorhizobium medicae* WSM419 uid58549
1419.5	2.378	3.889	*Haloterrigena turkmenica* DSM 5511
1115.8	2.111	3.911	Novosphingobium PP1Y uid67383
1425.8	2.452	3.926	Rhizobium NGR234 uid59081
1403.3	2.394	3.928	*Cupriavidus metallidurans* CH34 uid57815
385.4	1.374	4.171	*Sodalis glossinidius morsitans* uid58553
1346.8	2.200	4.369	Pantoea At 9b uid55845
1399.1	2.392	4.382	*Rhizobium etli* CFN 42 uid58377
1483	2.677	4.494	*Candidatus* Cloacamonas acidaminovorans Evry
1383.1	2.276	4.513	*Rhizobium etli* CIAT 652 uid59115
1402.4	2.501	4.538	*Rhizobium leguminosarum bv trifolii* WSM2304
1313.8	2.186	4.598	*Arthrobacter aurescens* TC1 uid58109
948.4	2.228	4.644	Mycobacterium JDM601 uid67369
1267	2.192	4.654	*Spirochaeta smaragdinae* DSM 11293 uid51369
1059.1	2.175	4.669	*Beutenbergia cavernae* DSM 12333 uid59047
1341.9	2.202	4.699	Arthrobacter FB24 uid58141
1430.8	2.614	4.767	*Rhizobium leguminosarum bv trifolii* WSM1325
1074.7	2.277	4.830	*Mycobacterium avium paratuberculosis* K 10
1328.2	2.229	4.972	Agrobacterium H13 3 uid63403
1345	2.241	4.986	Nocardioides JS614 uid58149
1331.3	2.247	5.010	*Agrobacterium vitis* S4 uid58249
1278	2.244	5.043	*Rhodococcus equi* 103S uid60171
1452	2.685	5.057	*Rhizobium leguminosarum bv viciae* 3841
1211.8	2.293	5.067	*Mycobacterium abscessus* uid61613
1368.9	2.274	5.200	Polaromonas JS666 uid58207
1277.9	2.287	5.288	*Bordetella petrii* uid61631
1268	2.334	5.339	*Bordetella bronchiseptica* RB50 uid57613
710.1	1.621	5.355	*Nostoc azollae* 0708 uid49725
1284.3	2.468	5.382	*Sphingomonas wittichii* RW1 uid58691
1074.9	2.495	5.475	*Mycobacterium avium* 104 uid57693
1275.8	2.399	5.548	*Mycobacterium gilvum* Spyr1 uid61403
1112.2	2.365	5.567	*Verminephrobacter eiseniae* EF01 2 uid58675
1303.6	2.491	5.620	*Mycobacterium gilvum* PYR GCK uid59421
1306.9	2.483	5.705	Mycobacterium MCS uid58465
1320.9	2.567	5.737	Mycobacterium KMS uid58491

1319.4	2.582	6.048	Mycobacterium JLS uid58489
1469.2	2.681	6.177	*Burkholderia phymatum* STM815 uid58699
860.6	1.827	6.196	*Pirellula staleyi* DSM 6068 uid43209
1465.6	2.866	6.283	*Streptomyces cattleya* NRRL 8057 DSM 46488
1402.8	2.761	6.359	*Conexibacter woesei* DSM 14684 uid43467
1435.8	2.700	6.516	*Rhodococcus erythropolis* PR4 uid59019
1471	2.940	6.558	*Cupriavidus necator* N 1 uid68689
1436.7	2.707	6.656	*Agrobacterium radiobacter* K84 uid58269
1449.2	2.938	6.988	*Mycobacterium smegmatis* MC2 155 uid57701
1403.9	2.779	7.013	*Achromobacter xylosoxidans* A8 uid59899
1464.6	2.952	7.097	*Pseudonocardia dioxanivorans* CB1190
1341.4	2.024	7.215	*Hahella chejuensis* KCTC 2396 uid58483
1057.6	1.961	7.750	*Trichodesmium erythraeum* IMS101 uid57925
1478.2	3.435	7.805	*Rhodococcus jostii* RHA1 uid58325
1474.4	3.344	7.913	*Rhodococcus opacus* B4 uid13791
1422.8	3.113	8.816	*Frankia EuI* 1c uid42615
1411	2.370	9.004	*Myxococcus fulvus* HW 1 uid68443
1319.3	2.349	9.446	*Haliangium ochraceum* DSM 14365 uid41425
1477.8	3.463	10.237	*Amycolatopsis mediterranei* U32 uid50565

[1] p.i.—paralog index, *Rank*—is an averaged rank calculated for multiple runs of the S-ranking procedure. Genomes are sorted by ascending size of genome for easier comparison with Figure 2.

Table S2. Complete list of atypical genomes according to *S-Rank*.

Rank	Size	Atypical Genomes	K	
622.8	1.591	*Candidatus* Korarchaeum cryptofilum OPF8	A	Crenarchaeota
791.4	1.782	*Thermofilum pendens* Hrk 5 uid58563	A	Crenarchaeota
792.6	1.842	*Thermoproteus tenax* Kra 1 uid74443	A	Crenarchaeota
808.9	1.846	*Thermococcus sibiricus* MM 739 uid59399	A	
754.3	1.908	*Pyrococcus furiosus* DSM 3638 uid57873	A	
804.9	1.936	*Thermoproteus uzoniensis* 768 20 uid65089	A	Crenarchaeota
803.4	2.001	*Halobacterium salinarum* R1 uid61571	A	
792.7	2.010	*Thermococcus barophilus* MP uid54733	A	
790.5	2.011	Thermococcus 4557 uid70841	A	
811.5	2.014	Halobacterium NRC 1 uid57769	A	
753.2	2.045	*Thermococcus gammatolerans* EJ3 uid59389	A	
875.7	2.046	*Ilyobacter polytropus* DSM 2926 uid59769	B	
797.8	2.121	*Pyrobaculum arsenaticum* DSM 13514 uid58409	A	
38.3	2.127	*Orientia tsutsugamushi* Boryong uid61621	B	
836.2	2.192	*Metallosphaera sedula* DSM 5348 uid58717	A	Crenarchaeota
1225.1	2.809	*Halalkalicoccus jeotgali* B3 uid50305	A	
1233.4	2.821	*Halogeometricum borinquense* DSM 11551	A	
1235.3	2.848	*Haloferax volcanii* DS2 uid46845	A	
1091.1	2.914	*Halophilic archaeon* DL31 uid72619	A	
1131.5	2.992	*Sulfolobus solfataricus* P2 uid57721	A	
1133.9	3.218	*Rhodobacter sphaeroides* ATCC 17025 uid58451	B	
1186.8	3.261	*Halorubrum lacusprofundi* ATCC 49239 uid58807	A	
206.9	3.268	*Mycobacterium leprae* Br4923 uid59293	B	
207.5	3.268	*Mycobacterium leprae* TN uid57697	B	
1417.6	3.311	Azospirillum B510 uid46085	B	
1240.8	3.420	*Haloarcula marismortui* ATCC 43049 uid57719	A	
1235	3.484	*Haloarcula hispanica* ATCC 33960 uid72475	A	
1236.4	3.521	*Ralstonia solanacearum* PSI07 uid50539	B	
1426.9	3.654	*Sinorhizobium meliloti* 1021 uid57603	B	
1306.5	3.668	*Halopiger xanaduensis* SH 6 uid68105	A	
1239	3.716	*Ralstonia solanacearum* GMI1000 uid57593	B	
1260.9	3.752	*Natrialba magadii* ATCC 43099 uid46245	A	
504.6	3.769	*Xanthomonas albilineans* GPE PC73 uid43163	B	

1401.9	3.782	*Sinorhizobium medicae* WSM419 uid58549	B
1419.5	3.889	*Haloterrigena turkmenica* DSM 5511 uid43501	A
1425.8	3.926	Rhizobium NGR234 uid59081	B
1403.3	3.928	*Cupriavidus metallidurans* CH34 uid57815	B
385.4	4.171	*Sodalis glossinidius morsitans* uid58553	B
1399.1	4.382	Rhizobium etli CFN 42 uid58377	B
1483	4.494	*Candidatus* Cloacamonas acidaminovorans Evry	B
666.6	4.627	*Leptospira interrogans serovar* Copenhageni Fiocruz	B
769.9	4.970	*Shewanella oneidensis* MR 1 uid57949	B
710.1	5.355	*Nostoc azollae* 0708 uid49725	B
852.8	5.473	*Isosphaera pallida* ATCC 43644 uid62207	B
860.6	6.196	*Pirellula staleyi* DSM 6068 uid43209	B
1057.6	7.750	*Trichodesmium erythraeum* IMS101 uid57925	B

Table S3. Paralogization indices of Shigella.

Rank	*Ave*	*Size*	*p.i.*	*mp*	**Genome**
893.2	1.80	4.6	0.26	0.43	*Shigella boydii* CDC 3083
875.9	1.82	4.5	0.26	0.42	*Shigella boydii* Sb227
879.9	1.90	4.4	0.25	0.39	*Shigella dysenteriae*
981.2	1.82	4.6	0.28	0.43	*Shigella flexneri* 2a 2457T
983.4	1.86	4.6	0.26	0.41	*Shigella flexneri* 2a 301
981.2	1.79	4.6	0.28	0.43	*Shigella flexneri* 5 8401
1008.1	1.88	4.8	0.28	0.43	*Shigella sonnei*

Table S4. Taxonomy of outliers.

a)

**Outliers in Figure 1**	
Taxa	Occurrence
Aliivibrio	1
Brucella	1
Candidatus	1
Ehrlichia	1
Mycobacterium	2
Orientia	1
Prevotella	1
Sodalis	1
Treponema	1
Vibrio	6

b)

**Outliers in Figure 2**			
Taxa	Occurrence	Taxa	Occurrence
Nostoc	1	Orientia	1
Achromobacter	1	Pantoea	1
Agrobacterium	3	Pirellula	1
Amycolatopsis	1	Polaromonas	1
Arthrobacter	2	Pseudonocardia	1
Azospirillum	1	Renibacterium	1
Beutenbergia	1	Rhizobium	6
Bordetella	2	Rhodococcus	4
Burkholderia	1	Sinorhizobium	2
Candidatus	1	Sodalis	1
Conexibacter	1	Sphingomonas	1
Cupriavidus	2	Spirochaeta	1
Frankia	1	Streptomyces	1

Hahella	1	Sulfolobus	1
Halobacteriaceae	9	Trichodesmium	1
Mycobacteriaceae	10	Verminephrobacter	1
Myxococcus	1		
Nocardioides	1		
Novosphingobium	1		
Phytoplasma	1		

c)

**Outliers in Figure 3**			
Taxa	Occurrence	Taxa	Occurrence
Nostoc	1	Pirellula	1
Azospirillum	1	Ralstonia	2
Candidatus Cloacamonas	1	Rhizobium	2
Candidatus Korarchaeum	1	Rhodobacter	1
Cupriavidus	1	Shewanella	1
Halobacteriaceae	12	Sinorhizobium	2
Ilyobacter	1	Sodalis	1
Isosphaera	1	Sulfolobus	1
Leptospira	1	Thermococcaceae	5
Metallosphaera	1	Thermofilum	1
Mycobacterium leprae	2	Trichodesmium	1
Orientia	1	Xanthomonas	1

d)

**Outliers in Figure 4**	
Taxa	Occurrence
Candidatus cloacamonas	1
Candidatus Ruthia	1
Gardnerella	1
Haemophilus	1
Mycoplasmataceae	8
Neisseria	5
Phytoplasmas	6
Staphylothermus	1
Streptococcus	1
Sulfolobus	2
Thermosphaera	1
Treponema	1

Table S6. *Orientia tsutsugamushi* and Rickettsias.

Rank	*Ave*	**Genome Size**	**# of Genes**	*p.i.*	*mp*	**Strain**
38.3	1.4	2.1	1182	0.09	0.36	*Orientia tsutsugamushi* Boryeong
66.8	1.8	2.0	1967[1]	0.12	0.42	*Orientia tsutsugamushi* Ikeda
60.2	1.2	1.2	1257	0.11	0.22	*Rickettsia akari* Hartford
81.1	1.3	1.5	1475	0.13	0.34	*Rickettsia bellii* OSU
103.8	1.3	1.5	1429	0.16	0.29	*Rickettsia bellii* RML369
40.6	1.1	1.2	1089	0.09	0.21	*Rickettsia canadensis* McKiel
101.1	1.2	1.3	1374	0.14	0.25	*Rickettsia conorii* Malish
124.2	1.4	1.5	1400	0.16	0.31	*Rickettsia felis*
63.1	1.2	1.3	1297	0.11	0.21	*Rickettsia heilongjiangensis*
67	1.1	1.3	971	0.10	0.21	*Rickettsia japonica*
67.9	1.2	1.4	968	0.10	0.25	*Rickettsia massiliae*
66.3	1.2	1.3	927	0.09	0.21	*Rickettsia peacockii* Rustic
60.2	1.1	1.1	843	0.09	0.22	*Rickettsia prowazekii* Madrid *E*
58.7	1.2	1.3	1343	0.11	0.21	*Rickettsia rickettsii* Sheila Smith
68.5	1.2	1.3	1382	0.12	0.22	*Rickettsia rickettsii* Iowa
56.8	1.1	1.1	837	0.09	0.21	*Rickettsia typhi* Wilmington

[1] The numbers of protein-coding genes are so different between two strains of *Orientia tsutsugamushi* as the annotation criteria were different between the two genomes.
